# Supplementary material for: Histone H3 serine-57 is a CHK1 substrate whose phosphorylation affects DNA repair
Source: Nat Commun. 2023 Aug 22;14:5104. doi: 10.1038/s41467-023-40843-4 (PMC10444856; doi:10.1038/s41467-023-40843-4)
Supplement: Supplementary file 1 — Supplementary Information [file 41467_2023_40843_MOESM1_ESM.pdf]

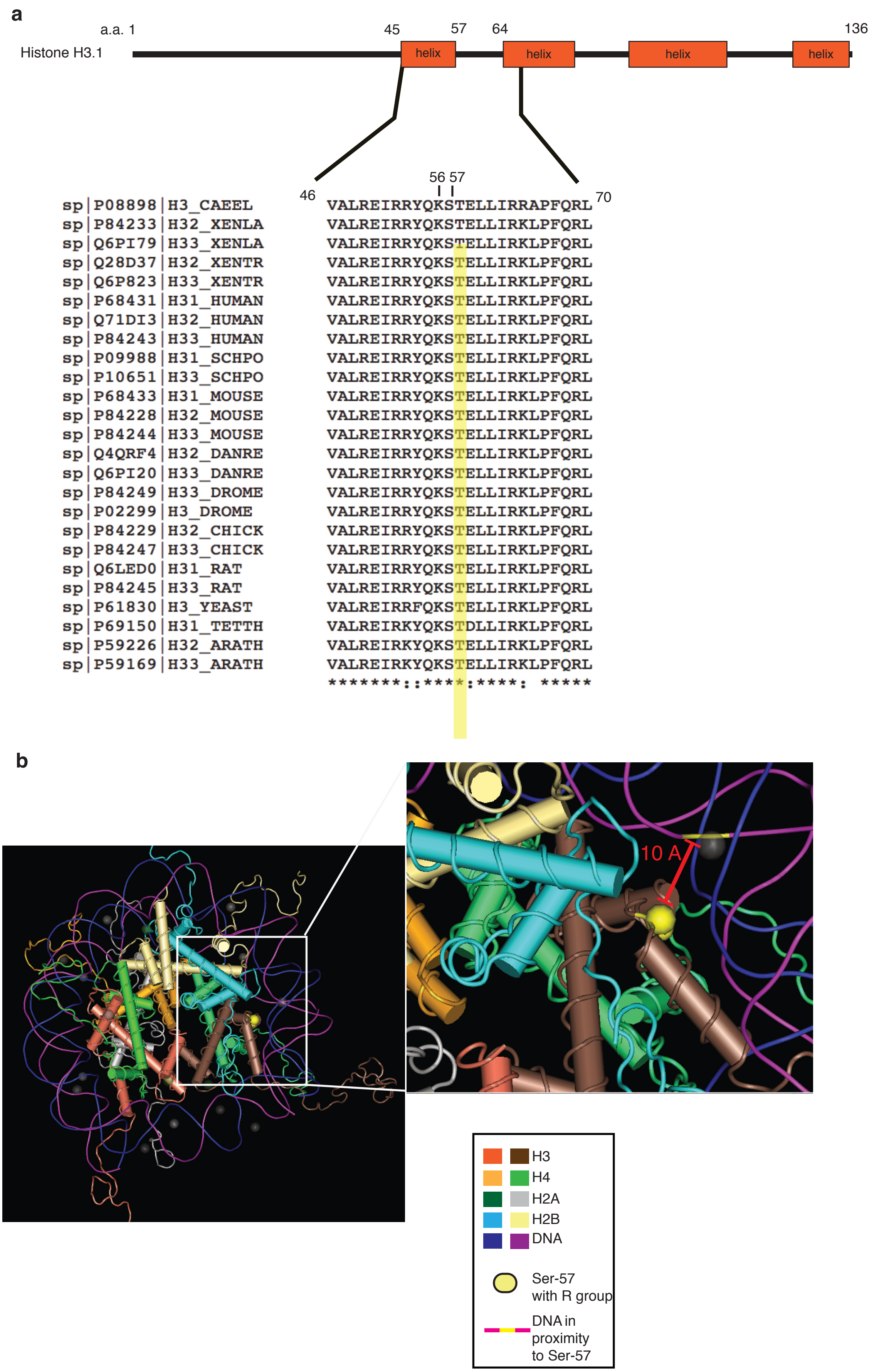

Supplementary Figure 1. Histone H3 serine-57 lies at the entry-exit point of the nucleosome. a) CRYSTALW multiple sequence alignment of histone H3 region of amino acids 46-70. b) 3D structure of the nucleosome with Ser-57 highlighted in yellow-coloured sphere from PDB file 1KX5.

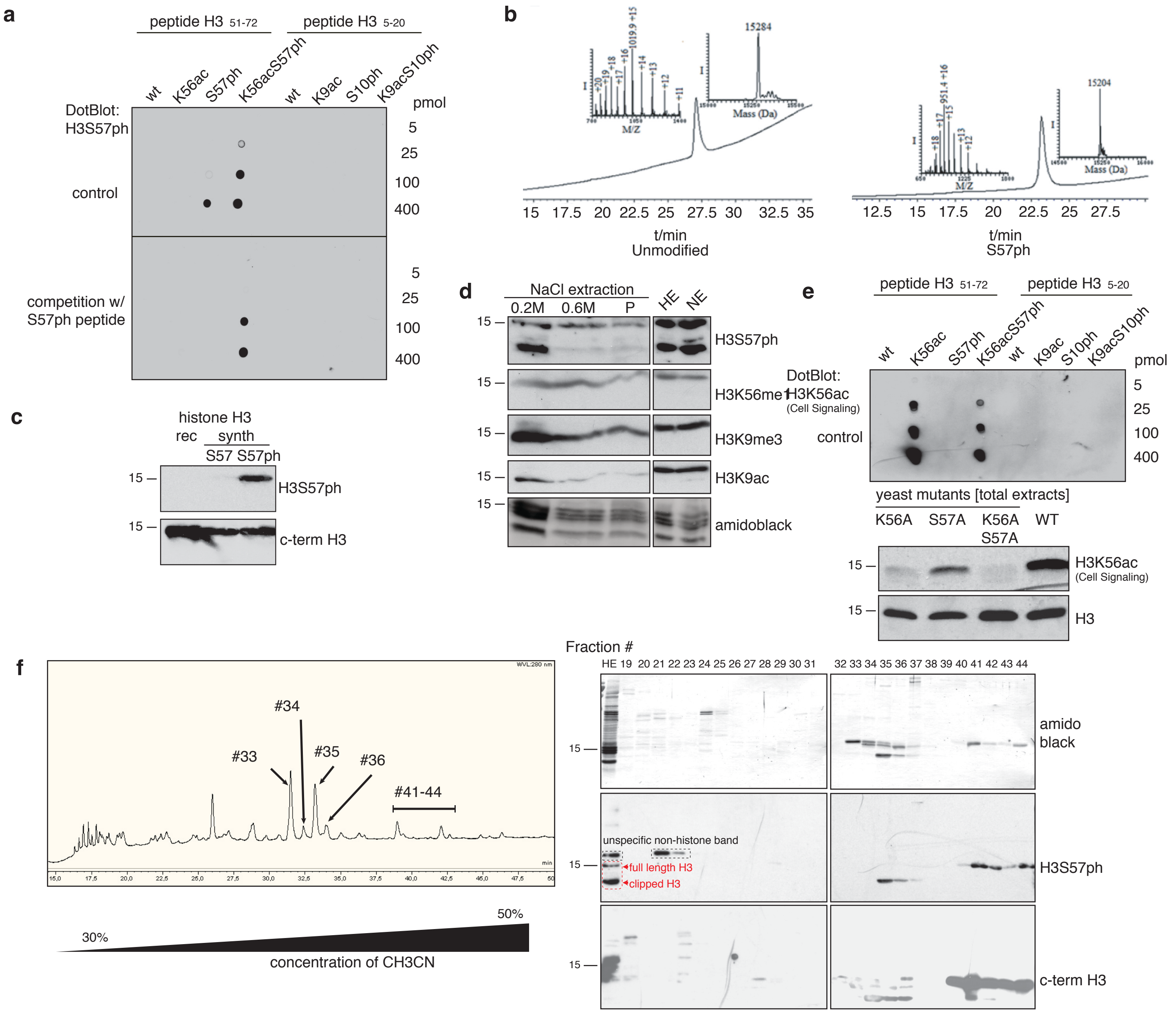

Supplementary Figure 2. Validation of H3S57ph and H3K56ac antibodies and identification of tailless histone H3 bearing both PTMs. a) Dot blots with synthetic H3 peptides bearing indicated PTMs, for our custom made H3S57ph antibody validation; n=3 independent experiments. b) Analytical HPLC and mass traces for purified H3S57ph (left) or unphosphorylated H3 (right) with the observed masses 15284 ± 1.1 Da (expected: 15283 Da) and 15204 ± 0.7 Da (expected: 15203 Da), respectively. c) Western blot analysis of anti-H3S57ph antibody against synthetised (synth) full-length histone H3 with Ser-57 phosphorylated (S57ph) or not (S57); rec, unmodified commercially available full-length H3 (Active Motif). d) WB of salt-extracted chromatin digested with micrococcal nuclease. Equal volumes from each salt fraction were analysed by 18% PAGE; 0.2M NaCl, mono-nucleosomes; 0.6M NaCl, oligo-nucleosomes; P, high-molecular weight chromatin pellet; NE, nuclear extract; HE, acid-extract histone extract; n=2 independent experiments. e) Top, dot blots as in (a), for a commercial H3K56ac antibody validation. Bottom, total cell extracts from *S. cerevisiae* cells expressing the indicated histone H3 mutants. f) Chromatogram of the separation of the acid-extracted histone fraction by HPLC and western blotting of collected fractions (18% PAGE). Note the ≈20 kDa band (fraction #21) that elutes from the C18 column at low acetonitrile concentrations indicating that it is an unspecific non-histone basic protein, hence ignored in the rest of the study. HE, histone extract before HPLC fractionation.

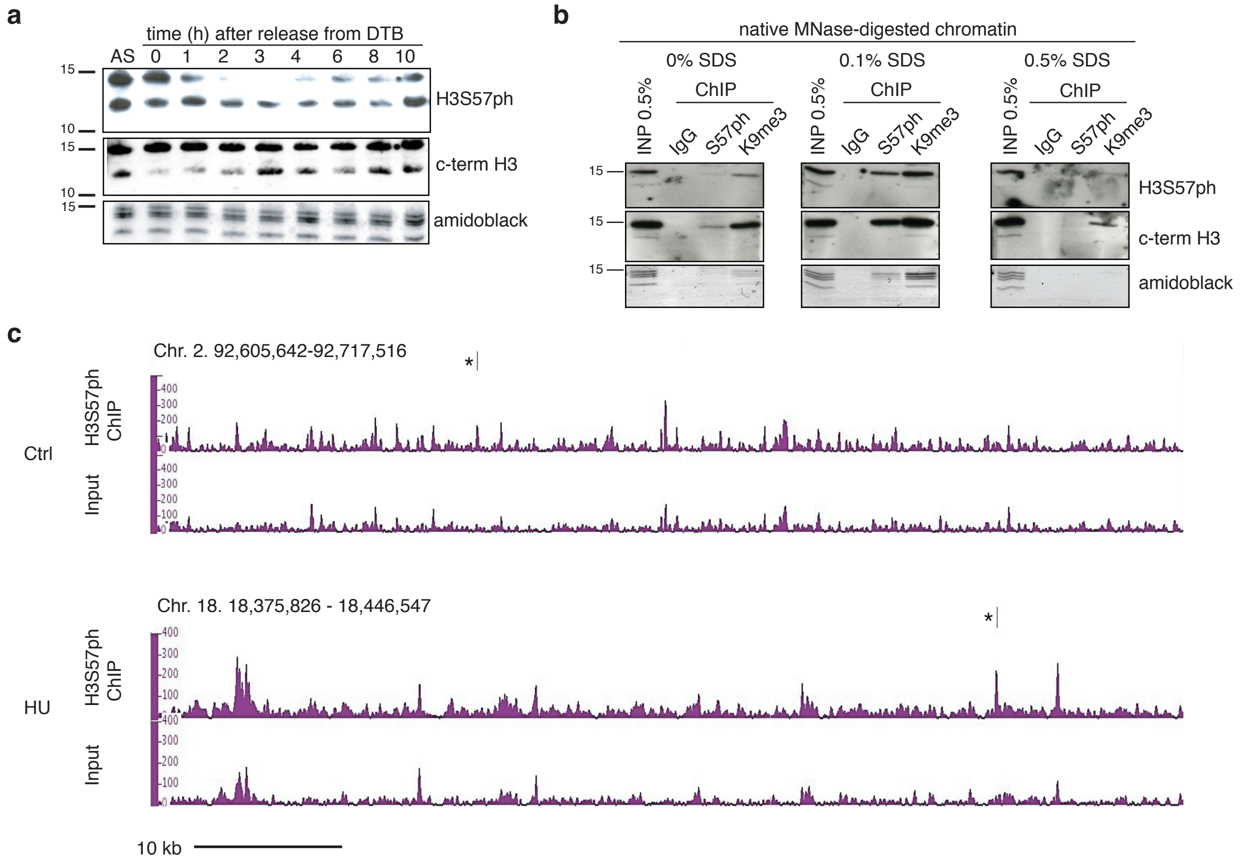

Supplementary Figure 3. H3S57ph does not show specific genomic localisation. a) WB (18% PAGE) of mouse ES cells synchronised by double thymidine block (DTB). b) ChIP-WB experiments to validate the efficiency of H3S57ph antibody in ChIP and to identify the optimal conditions. Pre-treatment (12 minutes) of native, MNase-digested nucleosomes with 0.1% SDS before final dilution and incubation with the antibody increased the immunoprecipitation of H3S57ph nucleosomes. H3K9me3 and H3 antibodies were used for positive controls; IgG beads for negative control; n=2 independent experiments. c) Examples of H3S57ph ChIP-seq data (two picks are indicated with asterisks) from U2OS cells in mock or HU-treated conditions.

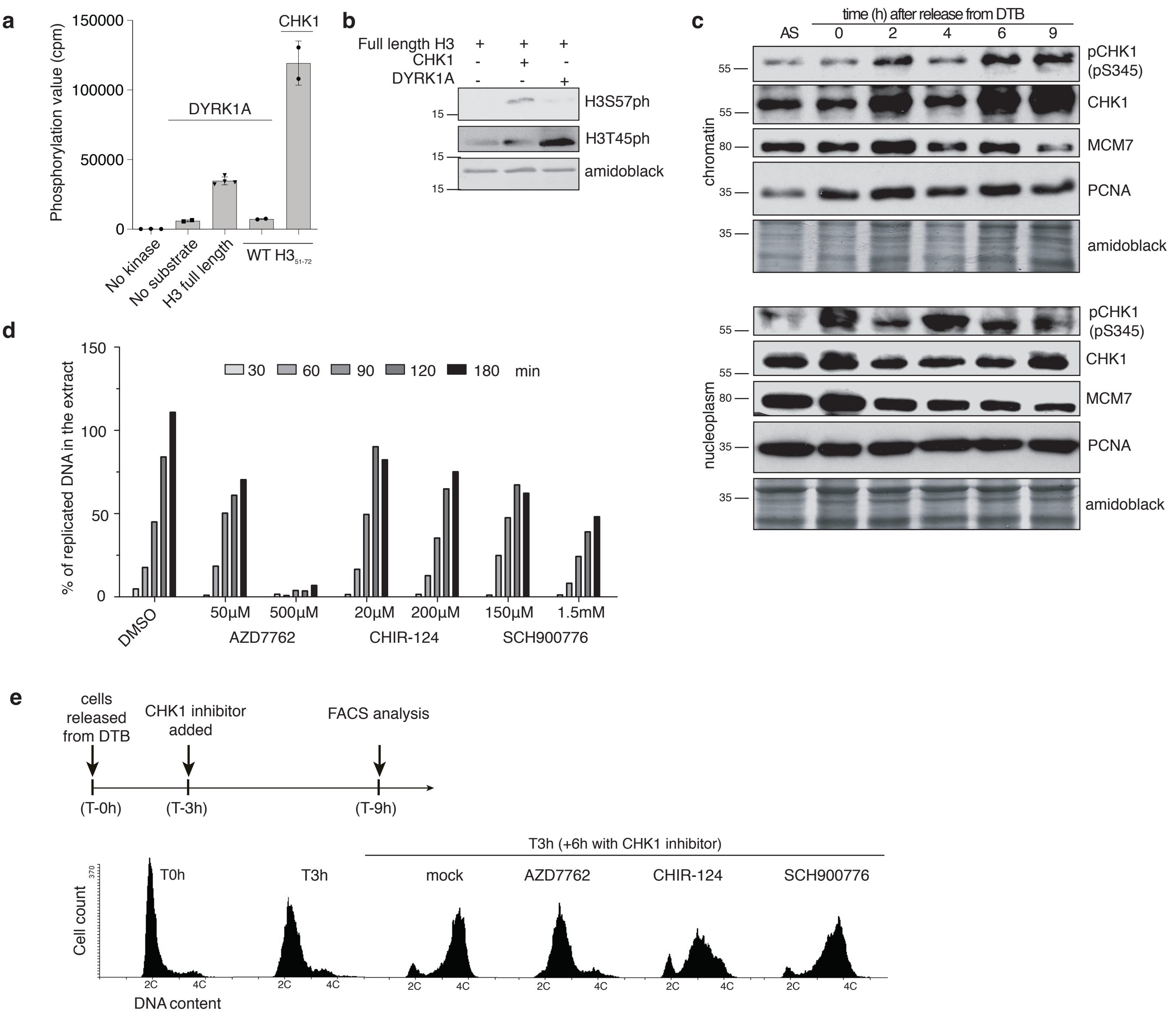

Supplementary Figure 4. H3S57ph is a direct target of CHK1. a) *In vitro* kinase assays using γ^{33}P-ATP, recombinant DYRK1A or CHK1 and full-length H3 or $H3_{51-72}$ peptide as substrate. Results represent scintillation counts; cpm, counts per minute; error bars, mean $\pm$ SD; n = 3. b) *In vitro* kinase assays as in (a) but only with non-radioactive ATP. Full-length H3 phosphorylation was analysed by western blotting. c) U2OS cells were synchronised at G1/S with double thymidine block (DTB), and chromatin and nucleoplasmic fractions were prepared from cells collected at the indicated time points after thymidine removal and analysed by WB for the proteins indicated. AS, asynchronous; amidoblack staining was used as loading control; n=2 independent experiments. d) DNA replication assay in *Xenopus* egg extracts in the presence of the indicated concentrations of three different Chk1 inhibitors; n=1 independent experiment. e) Top, scheme of the experiment and FACS profiles (bottom) of U2OS cells synchronised with double thymidine block (DTB), released for 3h and non-treated (mock) or treated with CHK1 inhibitors for 6h; n=2 independent experiments.

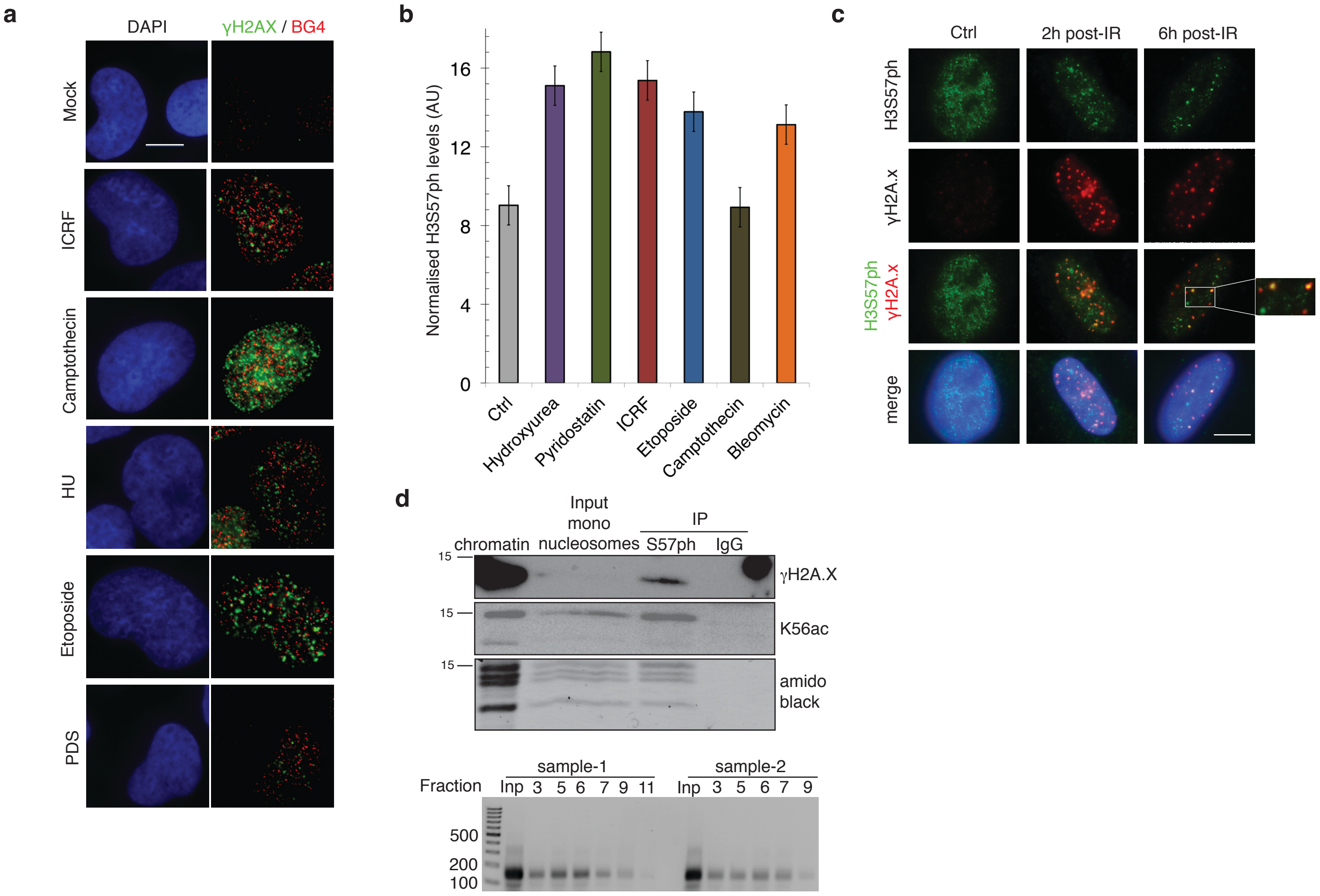

Supplementary Figure 5. H3S57ph is not a general marker of DNA damage. a) Immunofluorescence images of U2OS cells treated with DNA damage-inducing drugs (ICRF, ICRF-193,10µg/ml; Camptothecin, 0.5µM; HU, hydroxyurea, 2 mM; Etoposide, 5 µM; PDS, pyridostatin, 10 µM) for 24h and assessed for BG4 foci and γH2A.X. Scale bar, 10µm; n=2 independent experiments. b) Quantification of H3S57ph intensity signal in U2OS cells treated as in a); mean ± SD are shown; n=2 independent experiments. c) Confocal planes of irradiated (10 Gy) U2OS cells stained for H3S57ph and γH2A.X. DNA was visualised with DAPI; scale bar, 5µm. d) Top: immunoprecipitation (IP) with the H3S57ph antibody (or rabbit IgG for ctrl) from purified mononucleosomes and western blotting for γH2A.X and H3K56ac. Cells were first treated with HU (2mM, 2h) before native chromatin preparation. Total chromatin, MNase chromatin before mononucleosome purification. Bottom: nucleosome profiling for selected fractions obtained from sucrose gradient purification. Fractions 3-9 were pooled and used as input for mono-nucleosome immunoprecipitations; n=1 independent experiment.

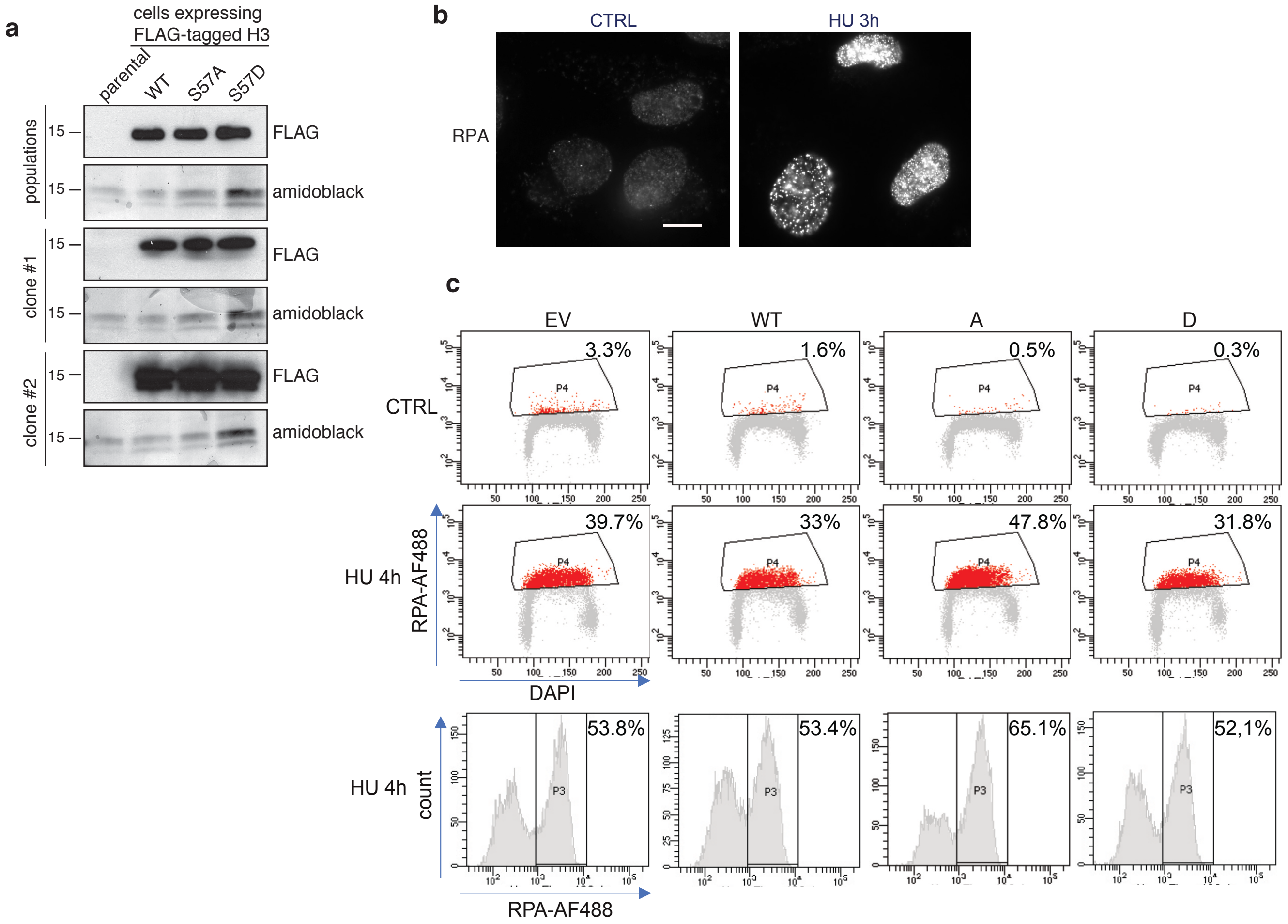

Supplementary Figure 6. Overexpressed H3S57 mutants alter responses to replication stress. a) Western blot analysis showing the expression levels of FLAG-tagged H3.1wt, S57A or S57D constructs under the CMV promoter in initial populations and two isolated clones (total cell extracts from U2OS cells). Parental is the original cell line before viral transfection; n=2 independent experiments. b) Immunoflurescence images of U2OS cells either in control conditions or treated with hydroxyurea (HU, 3h) and stained for RPA, to illustrate the increase in chromatin-bound RPA upon replication stress; scale bar, 10μm. c) Analysis of chromatin-bound RPA by FACS in non-treated cells, or cells treated with HU for 4h; DAPI was used to counterstain DNA; the fluorochrome used is indicated (AF, AlexaFluor). Doublets and debris were excluded; 10 000 cells were analysed per sample. Gates were arbitrary set in EV control conditions and implemented on the other conditions. The gating strategy is shown in the Supplementary Fig. 11. Representative images from 2 independent experiments are shown.

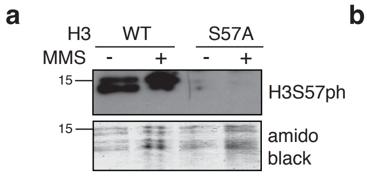

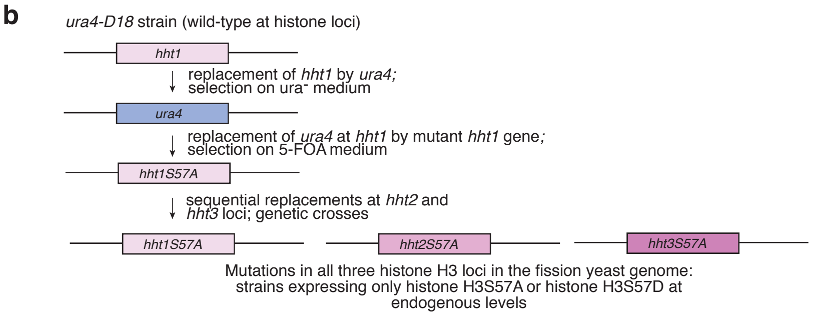

c

	Generation time (min)	Cell length at division (μm)	Septation index (%)
WT	139.87 ± 1.40	13.54 ± 0.19	14.51 ± 1.61
H3S57A	141.60 ± 1.84	13.81 ± 0.44	14.59 ± 1.70
H3S57D	143.42 ± 0.98	14.71 ± 0.40	14.81 ± 2.28

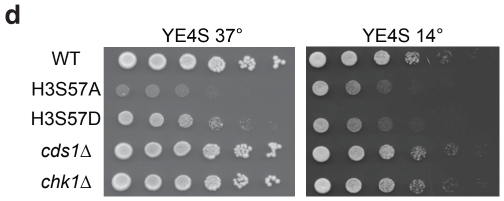

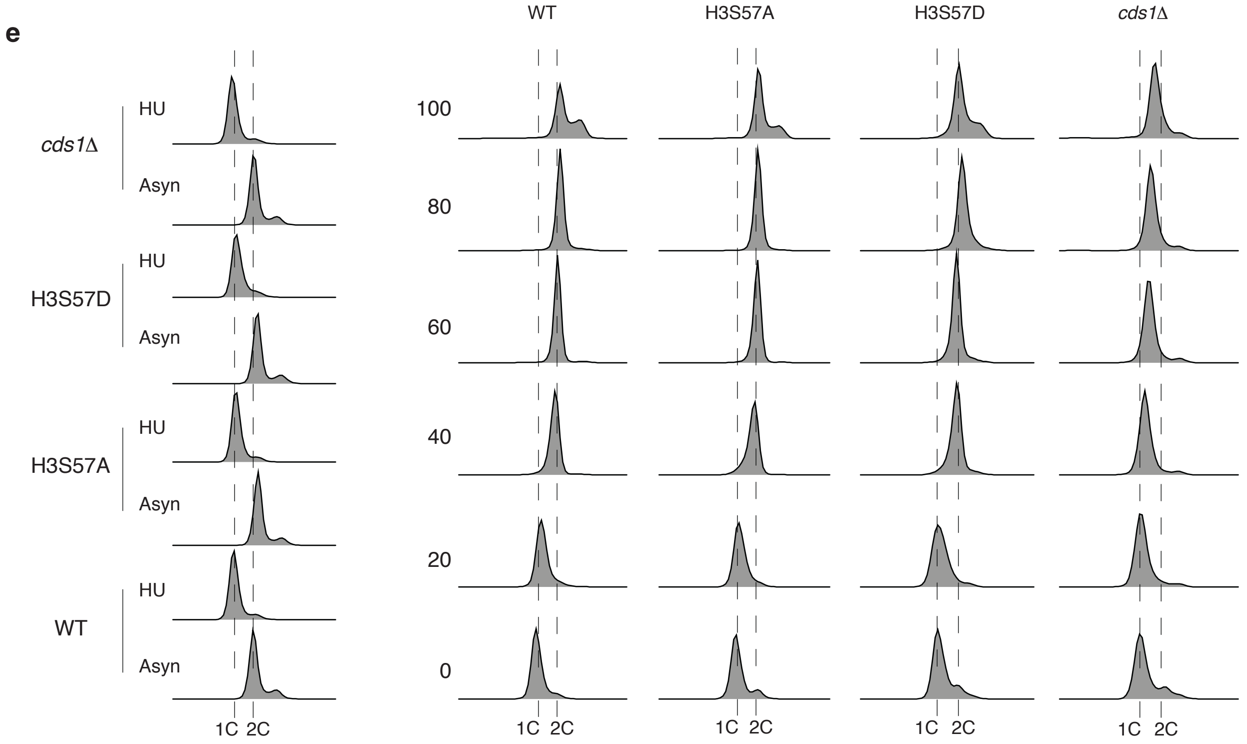

Supplementary Figure 7. H3S57ph is a modification conserved in fission and budding yeast, and is not required for growth in normal conditions. a) Western blot analysis (15% SDS-PAGE) of acid-extracted histones from *S. cerevisiae* encoding only WT or S57A mutant H3. MMS, methyl methane sulfonate at 0.01%; amidoblack staining was used as loading control. b) Illustration of the strategy for constructing full replacements of all three histone H3 loci in fission yeast with the indicated altered alleles. All proteins are expressed from their endogenous loci. c) Characterisation of fission yeast cells with the indicated genotypes. Averages and standard errors from three independent experiments are shown. d) Fission yeast cells with the indicated genotypes were grown to exponential phase in YE4S and spotted on plates at 10-fold dilutions. Images were taken on day 3 for YE4S 37ºC, and day 21 for YE4S 14ºC after spotting. e) Analysis of the wild-type and H3S57 mutant cells in the response to replication stress. Left, DNA content of proliferating cells and cells treated with 12 mM hydroxyurea (HU) for 4 hours. Right, cell cycle re-entry following HU arrest. DNA content was analysed at the indicated time points following removal of HU. In left and right, a strain lacking Cds1, the DNA replication checkpoint kinase and orthologue of human CHK1, was used as a control for cells defective for the replication stress response.

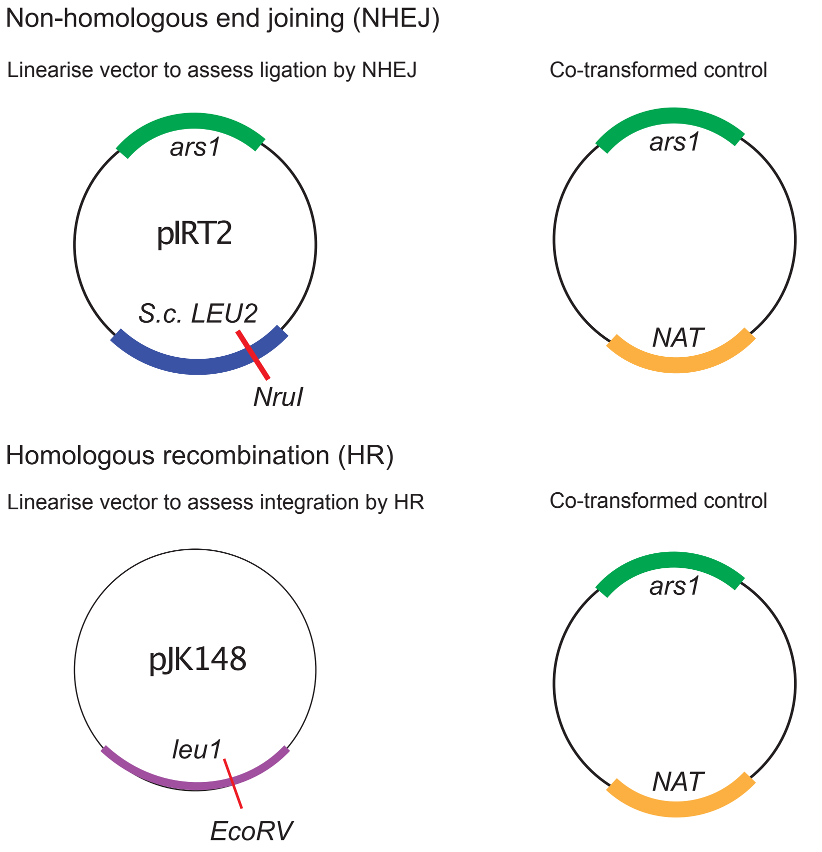

Supplementary Figure 8. Diagrams of the vectors used in the NHEJ and HR assays in fission yeast. Vectors for each assay were linearised at the indicated sites, purified, and co-transformed with the control vector to allow for internal normalisation.

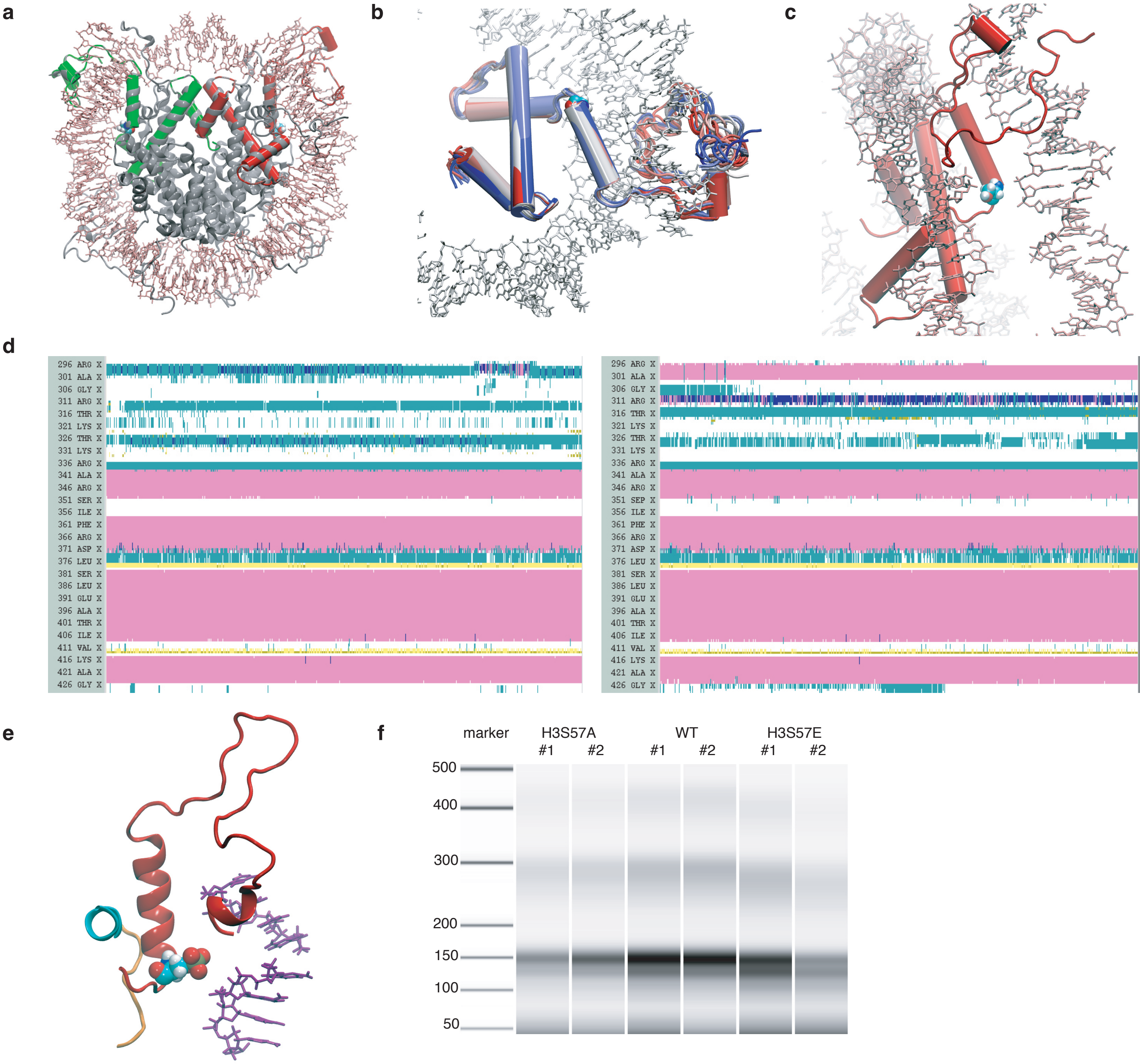

Supplementary Figure 9. H3S57ph affects nucleosome dynamics. a) Representation of the nucleosome core particle highlighting the H3 proteins in red and green, and S57 in VdW representation (final structure of the MD). b) Zoom on the H3 protein and the DNA strands in contact. H3 is represented from red (first structure of the MD), through white, to blue (last structure of the trajectory). c) Representation of the H3.1 protein in red, and the DNA in contact with H3 (in pink). d) Secondary structure map along time for residues 295 to 429 (H3) for unphosphorylated and H3S57ph nucleosomes, respectively. Helices are represented in blue and pink, turns in cyan, and random coils in white. Note the formation of two small helices (one blue, one pink) in the N-terminal region of H3S57ph. e) Residues within 5 Å of the H3S57 residue. H3 is represented in red, and DNA in violet. (f) BioAnalyzer gel showing MNase digestion pattern for the indicated samples from budding yeast strains expressing WT H3, or H3S57A or H3S57E mutants.

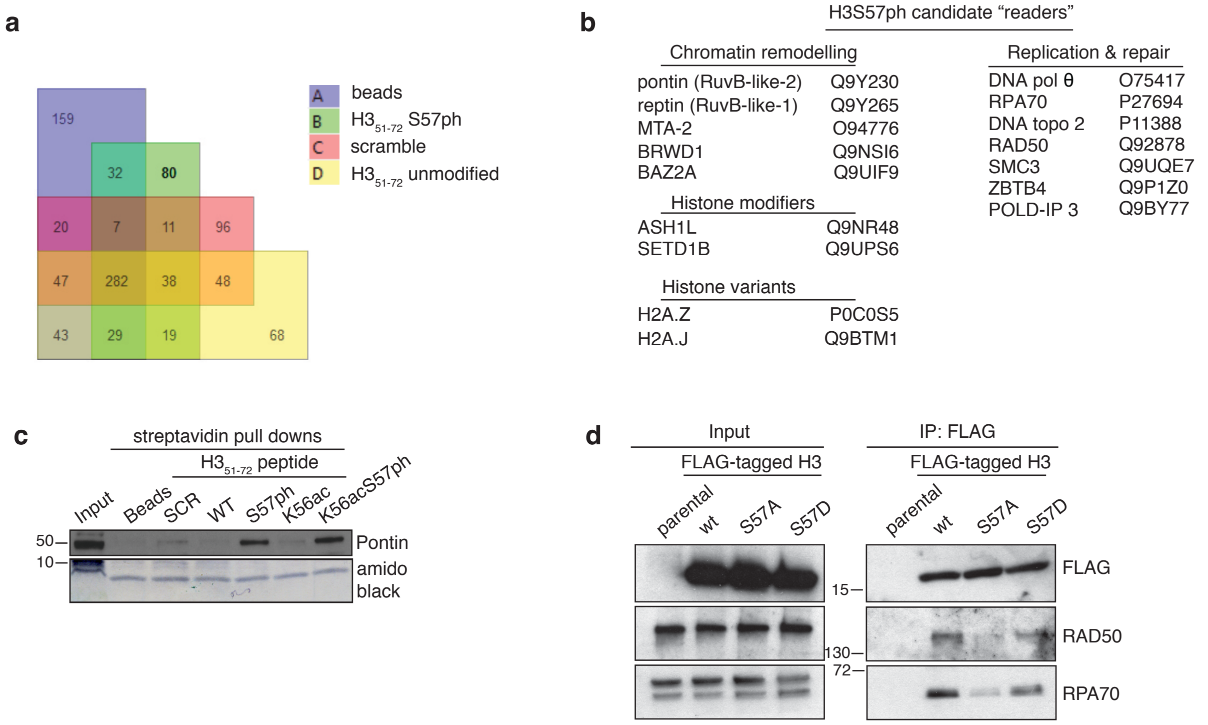

Supplementary Figure 10. HS57ph binds proteins with roles in chromatin remodeling and DNA repair. a) Venn diagram representing the numbers of proteins identified by mass spectrometry from the histone reader pull-down assay, in each MS/MS analysis. b) List of selected candidate H3S57ph "readers" and their Uniprot accession numbers. See Supplementary Dataset 3 for full results. c) Western blot analysis of the histone peptide pull-down assay confirming pontin as a candidate H3S57ph reader. d) WB of FLAG immunoprecipitations from U2OS clones expressing H3 WT or S57A/D mutants, after 1h recovery from HU treatment; n=2 independent experiments.

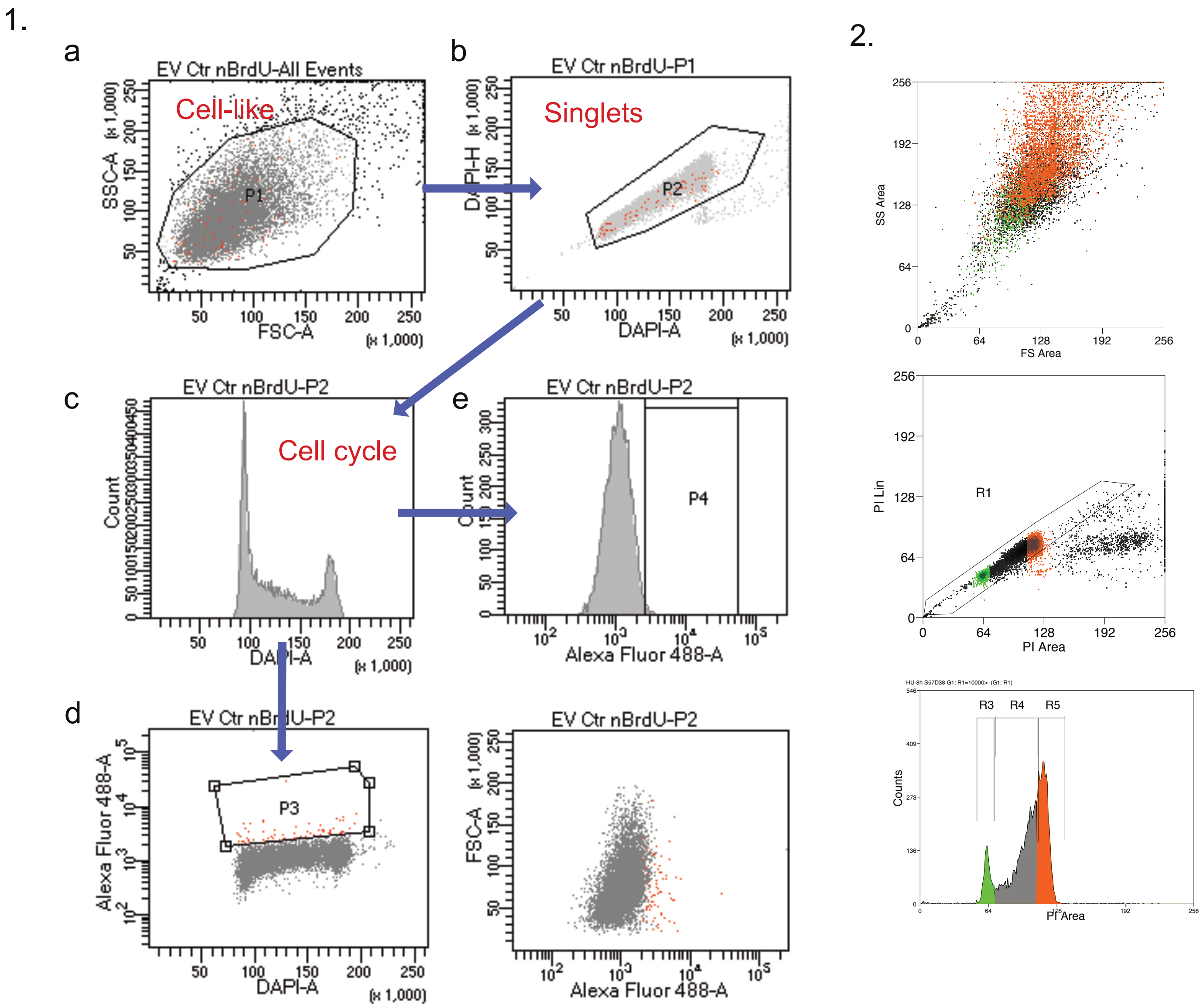

Tube: EV Ctr nBrdU

Population	#Events	%Parent	%Total
All Events	10,000	####	100.0
P1	9,380	93.8	93.8
P2	9,149	97.5	91.5
P3	67	0.7	0.7
P4	82	0.9	0.8

Supplementary figure 11. Flow cytometry gating strategy. 1. Gating strategy for Figures 4c-f and Supp Fig 6c. Gating hierarchy of a representative sample (here, empty-vector (EV)-expressing cells, control condition, stained for BrdU labelled with Alexa Fluor 488). From left to right, top to bottom: a) forward-scatter area linear scale (FSC-A) versus side-scatter area linear scale (SSC-A), identification of cell-like events; b) 405-nm laser area linear scale (DAPI-A) versus 405-nm laser hight linear scale (DAPI-H), isolation of single cells (singlets); c) DAPI-A versus counts, showing cell cycle profile; d) DAPI-A linear scale measuring DAPI content versus 488-nm laser area logarithmic scale (Alexa Fluor488-A), gating BrdU-positive cells. This strategy was used for Figures 4c-f and Supp Fig 6c, top. Further analysis in Supp Fig 6c, bottom, was done as in e), where a histogram plot was used to define the background signal, plotting 488-nm laser area logarithmic scale (Alexa Fluor488-A) against counts.
2. Gating strategy for Figures 2c, 3f, i; 4b, and Supp Fig 4e. Top, no gating. Middle, R1 gate represents single cells (plot of 405-nm laser area linear scale (PI-A) versus 405-nm laser hight linear scale (PI-H)). Bottom, cell cycle phases were assigned on PI-A versus counts. R3, G1 phase, R4, S-phase, R5, G2/M phase; PI, propidium iodide.

	WT-1	WT-2	H3S57A-1	H3S57A-2	H3S57E-1	H3S57E-2
Fuzzy	59.56	58.13	61.07	60.13	61.35	61.7
Uncertain	0.7	0.55	0.6	0.66	0.63	0.59
Well positioned	39.72	41.32	38.34	39.2	38.03	37.7

Supplementary Table 1. Summary of results of nucleosome mapping in yeast expressing different histone H3 alleles (2 biological replicates for each), showing percentages of fuzzy, uncertain or well-positioned nucleosomes from MNase sequencing.

Target	Source	Reference	Dilution WB	Dilution IF	Dilution FACS
γH2AX	Millipore	#05-636; clone JBW301	1:1000	1 :500	
γH2AX	Cell Signaling	#2577			1:200
c-term H3	Abcam	ab1791	1:5000		
H3K56me1	Active Motif	#39274	1:1000		
H3S10ph	Cell Signaling	#9706	1:1000		
H3T45ph	Active Motif	#39737	1:1000		
H3K9me3	Abcam	ab8898	1:1000		
H3K9ac	Upstate Millipore	#06-942	1:1000		
H3cs.1 cleavage-specific	D. Allis, Rockefeller University, N.Y., U.S.A.	doi: 10.1016/j.cell.2008.09.055	1:200		
Chk1	Cell Signaling	#2360S	1:1000		
phChk1	Cell Signaling	#2344 ; phS317	1:1000		
phChk1	Cell Signaling	#2348 ; phS345	1:1000		
Actin	Sigma	#A5441 ; clone AC-15	1:7500		
Pontin	M. Méchali, IGH, Montpellier, France		1:500		
Cyclin A	Santa Cruz Biotechnologies	sc-751; clone H-432	1:1000	1:250	
MCM7	Santa Cruz Biotechnologies	sc-71550	1:1000		
PCNA	Abcam	ab18197	1:1000		
FLAG	Sigma	M2 F3165	1:4000		
FLAG	Cell Signaling	#2368	1:1000		
RAD50	GenTex	GTX119731	1:1000		
RPA70	Abcam	ab79398	1:1000		
RPA2	Abcam	ab2175; clone 9H8		1:500	1:200
BrdU	BD Biosciences	#347580; clone B44			1:200
53BP1	Bio-Techne	NB100-304			1:200
AlexaFluor 546 conjugated goat anti-mouse	Invitrogen	A11003		1:1000	1:1000
AlexaFluor 488 conjugated goat anti-rat	Invitrogen	A11006		1:1000	1:1000
Goat anti-mouse IgG (H+L) HRP	Thermo Fisher	#32230	1:10000		
Goat anti-rabbit IgG (H+L) HRP	Thermo Fisher	#32260	1:10000		

Supplementary Table 2. List of commercial antibodies used in this study.

**Strain**	**Genotype**	**Source**
PN1	*h- 972*	Paul Nurse
JW1089	*h- cds1D::natMX6*	This study
JW1567	*h- hht1-S57A hht2-S57A hht3-S57A*	This study
JW1569	*h- hht1-S57D hht2-S57D hht3-S57D*	This study
JW1595	*h- rad52-GFP(S65T)::natMX6*	This study
JW1596	*h- hht1-S57A hht2-S57A hht3-S57A rad52-GFP(S65T)::natMX6*	This study
JW1597	*h- hht1-S57D hht2-S57D hht3-S57D rad52-GFP(S65T)::natMX6*	This study
JW1877	*h- hht1-S57A hht2-S57A hht3-S57A leu1-32*	This study
JW1879	*h+ hht1-S57D hht2-S57D hht3-S57D leu1-32*	This study
DC1	*h- leu1-32*	Damien Coudreuse
DC942	*rad52D::ura4+ leu1-32 h+*	Damien Coudreuse
DC981	*lig4D::kanMX6 leu1-32 h+*	Damien Coudreuse

Supplementary Table 3. List of fission yeast strains used in this study.
