# Supplementary material for: Histone H3 serine-57 is a CHK1 substrate whose phosphorylation affects DNA repair
Source: Nat Commun. 2023 Aug 22;14:5104. doi: 10.1038/s41467-023-40843-4 (PMC10444856; doi:10.1038/s41467-023-40843-4)
Supplement: Supplementary file 3 — Description of Additional Supplementary Files [file 41467_2023_40843_MOESM3_ESM.pdf]

Description of Additional Supplementary Files

Supplementary Data 1.
Proteomics data identifying peptides and their corresponding proteins on chromatin isolated from replicating *Xenopus* egg extracts.

Supplementary Data 2.
Kinase assay data from *in vitro* kinome screen using $H3_{51\text{-}72}$ peptide and 190 purified Ser/Thr kinases.

Supplementary Data 3.
Proteomics data identifying peptides and their corresponding proteins purified from biotin-labelled H3 peptide pulldowns from nuclear extracts of U2OS cells.

Supplementary Data 4.
Structure file (PDB) of representative structure of unmodified nucleosomes.

Supplementary Data 5.
Structure file (PDB) of representative structure of nucleosomes containing H3S57ph.
