# Supplementary material for: Histone H3 serine-57 is a CHK1 substrate whose phosphorylation affects DNA repair
Source: Nat Commun. 2023 Aug 22;14:5104. doi: 10.1038/s41467-023-40843-4 (PMC10444856; doi:10.1038/s41467-023-40843-4)
Supplement: Supplementary file 9 — Reporting Summary [file 41467_2023_40843_MOESM9_ESM.pdf]

nature portfolio

Corresponding author(s): Daniel Fisher

Last updated by author(s): 25/7/2023

# Reporting Summary

Nature Portfolio wishes to improve the reproducibility of the work that we publish. This form provides structure for consistency and transparency in reporting. For further information on Nature Portfolio policies, see our Editorial Policies and the Editorial Policy Checklist.

## Statistics

For all statistical analyses, confirm that the following items are present in the figure legend, table legend, main text, or Methods section.

| n/a | Confirmed | |
|---|---|---|
| ☐ | ☒ | The exact sample size (*n*) for each experimental group/condition, given as a discrete number and unit of measurement |
| ☐ | ☒ | A statement on whether measurements were taken from distinct samples or whether the same sample was measured repeatedly |
| ☐ | ☒ | The statistical test(s) used AND whether they are one- or two-sided<br>*Only common tests should be described solely by name; describe more complex techniques in the Methods section.* |
| ☐ | ☒ | A description of all covariates tested |
| ☒ | ☐ | A description of any assumptions or corrections, such as tests of normality and adjustment for multiple comparisons |
| ☐ | ☒ | A full description of the statistical parameters including central tendency (e.g. means) or other basic estimates (e.g. regression coefficient) AND variation (e.g. standard deviation) or associated estimates of uncertainty (e.g. confidence intervals) |
| ☐ | ☒ | For null hypothesis testing, the test statistic (e.g. *F*, *t*, *r*) with confidence intervals, effect sizes, degrees of freedom and *P* value noted<br>*Give P values as exact values whenever suitable.* |
| ☒ | ☐ | For Bayesian analysis, information on the choice of priors and Markov chain Monte Carlo settings |
| ☒ | ☐ | For hierarchical and complex designs, identification of the appropriate level for tests and full reporting of outcomes |
| ☒ | ☐ | Estimates of effect sizes (e.g. Cohen's *d*, Pearson's *r*), indicating how they were calculated |

*Our web collection on statistics for biologists contains articles on many of the points above.*

## Software and code

Policy information about availability of computer code

| | |
|---|---|
| Data collection | No software was used to collect data |
| Data analysis | Molecular dynamics simulations were carried out using the PMEMD CUDA code module of AMBER 14, and analyzed with CPPTRAJ, VMD, and CURVES+. References for these programs have been provided. Flow cytometry analysis was performed using either FacsCalibur BD, or LSR Fortessa Becton Dickinson, run by FACSDiVa software (version 9.0.1: Fig 4c-f and Supp 6c). FACS figures 2c and Supp 4e were prepared with Flowing Software (http://www.flowingsoftware.com/ - versions 2.4.1 and above), and Fig. 3f, i were prepared with FlowJo. Fission yeast analysis was performed using a BD Accuri C6 flow cytometer (BD Biosciences, Franklin Lakes, NJ, USA) and the FlowJo analysis software (FlowJo LLC, Ashland, OR, USA). ChIP-seq reads were aligned to the human genome using Bowtie2. Wig files were created using Pasha R package (Fenouil 2015). Statistical analysis was performed with Graphpad Prism 5. |

For manuscripts utilizing custom algorithms or software that are central to the research but not yet described in published literature, software must be made available to editors and reviewers. We strongly encourage code deposition in a community repository (e.g. GitHub). See the Nature Portfolio guidelines for submitting code & software for further information.

## Data

Policy information about availability of data

All manuscripts must include a data availability statement. This statement should provide the following information, where applicable:

- Accession codes, unique identifiers, or web links for publicly available datasets
- A description of any restrictions on data availability
- For clinical datasets or third party data, please ensure that the statement adheres to our policy

The proteomics data generated in this study have been deposited in the ProteomeXchange Consortium via the PRIDE partner repository with the dataset identifier PXD011328 and 10.6019/PXD011328 https://www.ebi.ac.uk/pride/).The MNase-seq data have been deposited in the Array Express under the accession number E-MTAB-6901. H3S57ph ChIP-seq data have been deposited in the GEO database, under the accession number GSE119742 (https://www.ncbi.nlm.nih.gov/geo/query/acc.cgi?acc=GSE119742). MD trajectories are publicly available through our BigNasim database (http://mmb.irbbarcelona.org/BIGNASim/).

## Human research participants

Policy information about studies involving human research participants and Sex and Gender in Research.

| | |
|---|---|
| Reporting on sex and gender | N/A |
| Population characteristics | N/A |
| Recruitment | N/A |
| Ethics oversight | N/A |

Note that full information on the approval of the study protocol must also be provided in the manuscript.

# Field-specific reporting

Please select the one below that is the best fit for your research. If you are not sure, read the appropriate sections before making your selection.

☒ Life sciences ☐ Behavioural & social sciences ☐ Ecological, evolutionary & environmental sciences

For a reference copy of the document with all sections, see nature.com/documents/nr-reporting-summary-flat.pdf

# Life sciences study design

All studies must disclose on these points even when the disclosure is negative.

| | |
|---|---|
| Sample size | This study did not involve experiments with living animals. Thus, it was not necessary to define sample sizes in advance to ensure adequate statistical power. |
| Data exclusions | No data were excluded from analysis |
| Replication | Our study conforms to the guidelines for statistics in cell biology described in Vaux, D.L. (2014) Basic statistics in cell biology. Annu Rev Cell Dev Biol 30, 23-37. Thus, most conclusions were drawn from independent lines of experiment rather than inferential statistics. All experiments were performed at least twice, and in most cases many more. Graphs without error bars (replication assays using Xenopus egg extracts) are presented in paired analysis where inter-group differences are important and reproducible, but inter-experimental variation across groups is large due to biological sensitivity of the assay. Here, representing the average and variance across experiments would obscure the intra-experimental difference between groups unless very large numbers of experiments were performed, which would be unfeasible. We do not present all replicates of the other experiments to avoid cluttering the paper with essentially duplicate data. Western blots and microscopy images are also representative of multiple experiments. Where individual cells or nuclei are shown, this is to better see their morphology, but they are representative of either the vast majority or all the cells/nuclei seen in the experiment. Where statistical tests are shown we report the method in the figure legend. |
| Randomization | No animals or patients that would require randomization were involved. |
| Blinding | Blinding was not relevant for this study as no data were generated by subjective human measurements. For western blots, FACS analysis, immunofluorescence, blinding is not possible because samples need to be loaded or analysed with knowledge of the sample identity. |

# Reporting for specific materials, systems and methods

We require information from authors about some types of materials, experimental systems and methods used in many studies. Here, indicate whether each material, system or method listed is relevant to your study. If you are not sure if a list item applies to your research, read the appropriate section before selecting a response.

## Materials & experimental systems

| n/a | Involved in the study |
|---|---|
| ☐ | ☒ Antibodies |
| ☐ | ☒ Eukaryotic cell lines |
| ☒ | ☐ Palaeontology and archaeology |
| ☒ | ☐ Animals and other organisms |
| ☒ | ☐ Clinical data |
| ☒ | ☐ Dual use research of concern |

## Methods

| n/a | Involved in the study |
|---|---|
| ☐ | ☒ ChIP-seq |
| ☐ | ☒ Flow cytometry |
| ☒ | ☐ MRI-based neuroimaging |

# Antibodies

Antibodies used

The anti-H3S57p antibody was produced by immunising two rabbits with the S57ph-containing peptide (IRRYQK{pSer}TELLIRKLPFQRLVR) (outsourced to Proteogenix). The collected sera were pooled and processed further to provide antibodies of highest specificity. Briefly, first, antibodies were affinity purified by bead-coupled S57ph peptide (as above) and depleted twice, first with the unmodified peptide (IRRYQKTELLIRKLPFQRLVR) and secondly with the N-tail peptide containing the S10ph that lies at a similar KST motif (QTAR(acet)-K{phSer}TGGKAPRKQL). The recovered purified antibody was diluted 1:1 with glycerol and stored at -20°C. Specificity was evaluated with budding yeast expressing only H3S57A, dot blots with synthetic peptides and peptide competitions, λ-phosphatase treatment as described in detail below. The antibody was used at 1:250 and 1:25 dilution for WB and IF, respectively.
The specific anti-H3K56ac antibody that we validated and used exclusively was from Cell Signaling (#4243). Other H3K56ac antibodies that we found to be less specific were: 39282 and 61062 from Active Motif, and 04-1135 from Millipore.
Other commercial antibodies used in human cells were:
Target Source Reference Dilution WB Dilution IF Dilution FACS
γH2AX Millipore #05-636; clone JBW301 1:1000 1 :500
γH2AX Cell Signaling #2577 1:200
c-term H3 Abcam ab1791 1:5000
H3K56me1 Active Motif #39274 1:1000
H3S10ph Cell Signaling #9706 1:1000
H3T45ph Active Motif #39737 1:1000
H3K9me3 Abcam ab8898 1:1000
H3K9ac Upstate Millipore #06-942 1:1000
H3cs.1 cleavage-specific D. Allis, Rockefeller University, N.Y., U.S.A. doi: 10.1016/j.cell.2008.09.055 1:200
Chk1 Cell Signaling #2360S 1:1000
phChk1 Cell Signaling #2344 ; phS317 1:1000
phChk1 Cell Signaling #2348 ; phS345 1:1000
Actin Sigma #A5441 ; clone AC-15 1:7500
Pontin M. Méchali, IGH, Montpellier, France 1:500
Cyclin A Santa Cruz Biotechnologies sc-751; clone H-432 1:1000 1:250
MCM7 Santa Cruz Biotechnologies sc-71550 1:1000
PCNA Abcam ab18197 1:1000
FLAG Sigma M2 F3165 1:4000
FLAG Cell Signaling #2368 1:1000
RAD50 GenTex GTX119731 1:1000
RPA70 Abcam ab79398 1:1000
RPA2 Abcam ab2175; clone 9H8 1:500 1:200
BrdU BD Biosciences #347580; clone B44 1:200
53BP1 Bio-Techne NB100-304 1:200
AlexaFluor 546 conjugated goat anti-mouse Invitrogen A11003 1:1000 1:1000
AlexaFluor 488 conjugated goat anti-rat Invitrogen A11006 1:1000 1:1000
Goat anti-mouse IgG (H+L) HRP Thermo Fisher #32230 1:10000
Goat anti-rabbit IgG (H+L) HRP Thermo Fisher #32260 1:10000

The BG4 probe was purified from the pSANG10-3F-BG4 (#55756; Addgene48) using standard protocols. Briefly, a clone of Rosetta bacteria transformed with the plasmid was cultured in 1L LB+kanamycin medium. At 0.6 OD600, 1 M IPTG was added and grown at 28°C to induce protein expression overnight (14 h). Expression was confirmed by SDS-PAGE. The bacterial pellet was resuspended in TES buffer (0.2 M Tris pH 8.0, 0.5 mM EDTA, 0.5 M sucrose) and incubated on ice with light shaking at which time point 18 ml TES/4 (1/4 diluted TES) and incubated for an additional hour. The lysate containing the periplasmic material was cleared by centrifugation (8000 rpm for 30 min). TALON beads (2 ml) were pre-washed with 5x bead volume of PBS and incubated overnight with the protein. Beads were washed with 10x volume (PBS with 5 mM imidazole). BG4 was eluted with PBS+500 mM imidazole. Imidazole was removed by successive rounds of dialysis in 3 L of PBS (3x1L, the second round was overnight). The purity and abundance of the purified probe was estimated by SDS-PAGE. Subsequently, the BG4 probe was diluted with 50% final glycerol and stored at -20°C.

Validation

Specificity of H3S57ph and H3K56ac was evaluated with yeast expressing only H3 alleles with these amino acids mutated, dot blots


March 2021

| | |
|---|---|
| Validation | with synthetic peptides and peptide competitions, and (H3S57ph only) λ-phosphatase treatment. All commercial antibodies used in this study are available and extensively validated by the company and others. Validation data is available in each of these company's website. These antibodies (with the exception of Histone H3K56ac, as described above and in the paper) were not directly validated by us but were validated by the manufacturer for the same species and applications as they were used in this study. |

# Eukaryotic cell lines

Policy information about cell lines and Sex and Gender in Research

| | |
|---|---|
| Cell line source(s) | U2OS were obtained from the ATCC. RPE, HeLa, MDA-MB-231, MCF10, PBMC, Jurkatt, KG1a, MV-4.11, CN-2, LNCaP and HEK293T cells were a gift from A. Castro lab (CRBM, Montpellier, France) and described in Vera et al., 2015. |
| Authentication | Cell lines from ATCC were authenticated by ATCC; no cell lines were authenticated by us. |
| Mycoplasma contamination | The absence of mycoplasma contamination was confirmed by weekly testing in the IGMM facility (Mycoalert kit) |
| Commonly misidentified lines (See ICLAC register) | No commonly misidentified cell lines (as defined by version 8.0 of ICLAC register) were used |

# ChIP-seq

## Data deposition

☒ Confirm that both raw and final processed data have been deposited in a public database such as GEO.

☒ Confirm that you have deposited or provided access to graph files (e.g. BED files) for the called peaks.

| | |
|---|---|
| Data access links *May remain private before publication.* | S57ph ChIP-seq data have been deposited in the GEO database, under the accession number GSE119742 (https://www.ncbi.nlm.nih.gov/geo/query/acc.cgi?acc=GSE119742). |
| Files in database submission | for GEO: ChIP-seq data: files:<br>S57-Input_U2OS_ctrl-1_R1.fastq<br>S57-Input_U2OS_ctrl-1_R2.fastq<br>S58-Input_U2OS_ctrl-2_R1.fastq<br>S58-Input_U2OS_ctrl-2_R2.fastq<br>S59-Input_U2OS_HU2mM2h-1_R1.fastq<br>S59-Input_U2OS_HU2mM2h-1_R2.fastq<br>S60-Input_U2OS_HU2mM2h-2_R1.fastq<br>S60-Input_U2OS_HU2mM2h-2_R2.fastq<br>S63-ChIP_U2OS_ctrl_H3S57p_1-RIPA_R1.fastq<br>S63-ChIP_U2OS_ctrl_H3S57p_1-RIPA_R2.fastq<br>S64-ChIP_U2OS_ctrl_H3S57p_2-RIPA_R1.fastq<br>S64-ChIP_U2OS_ctrl_H3S57p_2-RIPA_R2.fastq<br>S65-ChIP_U2OS_HU_H3S57p_1-RIPA_R1.fastq<br>S65-ChIP_U2OS_HU_H3S57p_1-RIPA_R2.fastq<br>S66-ChIP_U2OS_HU_H3S57p_2-RIPA_R1.fastq<br>S66-ChIP_U2OS_HU_H3S57p_2-RIPA_R2.fastq<br>WIGfs_S57-Input_U2OS_ctrl-1_mergedReads_elPairs-Est158_AThr7_bin50.wig<br>WIGfs_S58-Input_U2OS_ctrl-2_mergedReads_elPairs-Est164_AThr7_bin50.wig<br>WIGfs_S59-Input_U2OS_HU2mM2h-1_mergedReads_elPairs-Est152_AThr6_bin50.wig<br>WIGfs_S60-Input_U2OS_HU2mM2h-2_mergedReads_elPairs-Est152_AThr5_bin50.wig<br>WIGfs_S63-ChIP_U2OS_ctrl_H3S57p_1-RIPA_mergedReads_elPairs-Est164_AThr7_bin50.wig<br>WIGfs_S64-ChIP_U2OS_ctrl_H3S57p_2-RIPA_mergedReads_elPairs-Est176_AThr8_bin50.wig<br>WIGfs_S65-ChIP_U2OS_HU_H3S57p_1-RIPA_mergedReads_elPairs-Est156_AThr6_bin50.wig<br>WIGfs_S66-ChIP_U2OS_HU_H3S57p_2-RIPA_mergedReads_elPairs-Est155_AThr7_bin50.wig |
| Genome browser session (e.g. UCSC) | No longer applicable |

## Methodology

| | |
|---|---|
| Replicates | 2 biological replicates were performed for each experimental point. |
| Sequencing depth | Reads were paired end 100 bases. A total of 206 million reads were obtained for the eight samples (from 18.4M to 29.1M per sample). Between 81.7 and 84.8% of reads aligned to a unique site, while 14.7-17.7% of reads aligned using multi-mapping. Total aligned reads were between 99.1 and 99.9% for different samples. |
| Antibodies | Antibodies used were H3S57ph (described in this paper) |
| Peak calling parameters | After filtering and quality control, reads were aligned to the human genome (version UCSC hg38) using Bowtie2. Wig files were created using Pasha R package (Fenouil 2015). Peak calling used MACS2 with default parameters. |
| Data quality | QC used the FastQC program. A total of 272 peaks were obtained |

| | |
|---|---|
| Software | QC used the FastQC program. Reads were aligned to the human genome (version UCSC hg38) using Bowtie2. Wig files were created using Pasha R package (Fenouil 2015). Peak calling used MACS2 with default parameters. |

# Flow Cytometry

## Plots

Confirm that:

☒ The axis labels state the marker and fluorochrome used (e.g. CD4-FITC).

☒ The axis scales are clearly visible. Include numbers along axes only for bottom left plot of group (a 'group' is an analysis of identical markers).

☒ All plots are contour plots with outliers or pseudocolor plots.

☒ A numerical value for number of cells or percentage (with statistics) is provided.

## Methodology

| | |
|---|---|
| Sample preparation | Cells were fixed in 70% ethanol, rehydrated in PBS and incubated with propidium iodide and RNase A as described in the reference provided |
| Instrument | Flow cytometry used a LSR Fortessa BD instrument for experiments shown in Fig 4c-f and Supplementary Fig 6c,, a CyAn ADP 9C for experiments in fig. 4b, a BD Accuri C6 flow cytometer for experiments shown in Supplementary Fig 7e, and FacsCalibur BD instrument for all other experiments |
| Software | Fig. 4b and Supplementary Fig. 7e, were prepared with FlowJo, Fig 4c-f and Supplementary Fig 6c using BD FACSDiva version 9.0.1, and all other FACS figures were prepared with Flowing Software (http://www.flowingsoftware.com/ - versions 2.4.1 and above), |
| Cell population abundance | 10000 |
| Gating strategy | Cells were gated on forward and side scatter to remove debris and dead cells. Further gating strategy for analysis of chromatin-bound proteins  is provided as a Supplementary figure. |

☒ Tick this box to confirm that a figure exemplifying the gating strategy is provided in the Supplementary Information.
